# An Italian Case of Disseminated Histoplasmosis Associated with HIV

**DOI:** 10.1155/2019/7403878

**Published:** 2019-11-16

**Authors:** Chiara Papalini, Barbara Belfiori, Giovanni Martino, Rita Papili, Lucia Pitzurra, Stefano Ascani, Maria Bruna Pasticci

**Affiliations:** ^1^Infectious Diseases Clinic, Perugia University, S. Maria della Misericordia Hospital, Perugia 06132, Italy; ^2^Hematology Clinic, Perugia University, S. Maria della Misericordia Hospital, Perugia 06132, Italy; ^3^Microbiology Institute, Perugia University, S. Maria della Misericordia Hospital, Perugia 06132, Italy

## Abstract

*Histoplasma capsulatum* is a dimorphic fungus, endemic in the Americas, Africa (var. *duboisii*), India, and Southeast Asia. *H. capsulatum* infection is rarely diagnosed in Italy, while in Latin America, progressive disseminated histoplasmosis (PDH) is one of the most frequent AIDS-defining illnesses and causes of AIDS-related deaths. We report a case of PDH and new HIV infection diagnosis in a Cuban patient, who has been living in Italy for the past 10 years. Bone marrow aspirate and peripheral blood smear microscopy suggested *H. capsulatum* infection. The diagnosis was confirmed with the culture method identifying its thermal dimorphism. Liposomal amphotericin B was administered alone for 10 days and then for another 2 days, accompanied with voriconazole; the former was stopped for probable side effects (persistent fever and worsening thrombocytopenia), and voriconazole was continued to complete 4 weeks. PDH maintenance treatment consisted of itraconazole for one year. Antiretroviral therapy (ART) was started on the third week of antifungal treatment. At the 3-year follow-up, the patient is adherent on ART, the virus was suppressed, and she has an optimal immune recovery. This case highlights the need to suspect histoplasmosis in the differential diagnosis of opportunistic infections in immunocompromised persons, native to or who have traveled to endemic countries.

## 1. Introduction

*H. capsulatum* is a dimorphic fungus that changes its features from mold to yeast depending on the temperature. It grows as a filamentous fungus in the soil, and the best range of temperature for the *H. capsulatum* growth is 25–28°C, developing macroconidia and microconidia. Inside the alveoli, microconidia germinate into the yeast phase (>35°C) within 3–5 days from the exposition. Neutrophils are parasitized in the first 24 h; then, *H. capsulatum* proliferates within macrophages and spreads into the host throughout them. The development of specific T-cell immunity is important to clear the infection after about 21 days [1].

Clinical manifestations range from asymptomatic pulmonary involvement up to disseminated forms with an acute or chronic onset. A deficit of cell-mediated immunity, such as in HIV/AIDS persons, often leads to disseminated disease.

Histoplasmosis in all its clinical manifestations is an underdiagnosed disease even in endemic regions [2].

In Italy, the occurrence of autochthonous cases of histoplasmosis is documented [3]. Nevertheless, the great majority of the cases are subjects native to or traveling in endemic areas [3–5].

Here, we report on a case of PDH and a new HIV diagnosis in a patient native to Cuba, who has been living in Italy for the past 10 years. The patient, 4 months before hospital admission, had spent a month in Cuba.

## 2. Case Presentation

In 2016, a 33-year-old female was admitted with fever (40°C) and maculopapular cutaneous lesions involving the face and the trunk. The patient referred that these symptoms had started one month prior. The patient, native to Cuba (Camaguey Region), has been living in Italy for the last decade, and 4 months prior to admission, she had traveled to Cuba for a month. She did not report any past medical conditions, but she did have unprotected sex in the past. HIV serology resulted positive, HIV-RNA 2,150,000 copies/ml, the CD4+ count evidenced 2/*μ*L cells (1%), and the CD4+/CD8+ ratio was zero. Empirical antibiotic therapy was started with piperacillin/tazobactam. Chest tomography (CT) detected a ground-glass pattern involvement of the lung parenchyma, multiple bilateral micronodules, mediastinal, supraclavicular, and lateral cervical lymphadenopathy. Suspecting *Pneumocystis jirovecii* pneumonia, the patient was referred to our Infectious Diseases Clinic.

**Figure 1:**
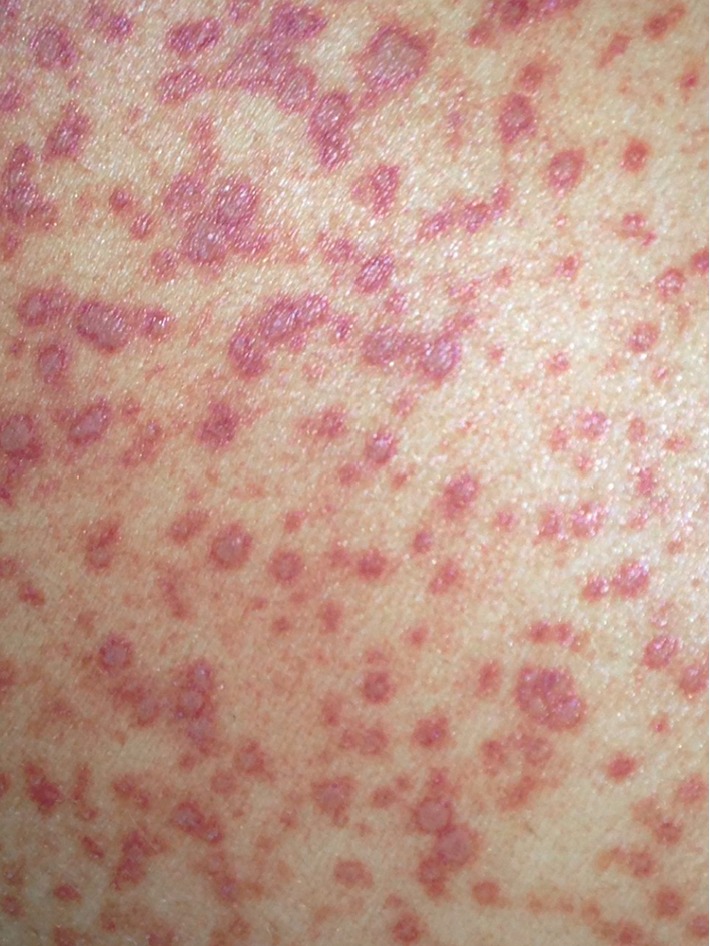
Maculopapular cutaneous lesions on the trunk.

Physical examination revealed fever (40°C), tachycardia (113 bpm), and tachypnea (40 breaths/min). Blood gas analysis on room air resulted in a pH of 7.49, pO_2_ of 70 mmHg, and pCO_2_ of 29 mmHg. Maculopapular cutaneous lesions were present on the face, trunk, and limbs (Figure 1); hepatosplenomegaly, lateral cervical lymphadenopathy, and fine crackles on both basal lung fields were also detected.

Laboratory findings showed pancytopenia (white blood cells 1 × 10^3^/*μ*L, platelets 137 × 10^3^/*μ*L, and haemoglobin 7.7 g/dL), alanine aminotransferase (ALT) 67 IU/L (normal value 0–45 IU/L), aspartate aminotransferase (AST) 446 IU/L (normal value 0–45 IU/L), gamma-glutamyl transferase (GGT) 147 IU/L (normal value 7–49 IU/L), alkaline phosphatase (ALP) 500 IU/L (normal value 80–320 IU/L), lactic dehydrogenase (LDH) 6752 IU/L (normal value 225–450 IU/L), gamma-globulin level 21%, C-reactive protein (C-RP) 24 mg/dL (normal value ≤ 0.5 mg/dL), ferritin >7500 ng/mL (normal value 11–307 ng/mL), and triglycerides 247 mg/dL (40–165 mg/dL). Routine blood and urine cultures, serology for syphilis, toxoplasma, and *Leishmania*, latex for *Cryptococcus neoformans*, and smear test for malaria resulted negative. Plasma *Cytomegaloviru*s-DNA was positive with 83087 copies/mL, as well as *Cytomegaloviru*s p65 antigen with 3 cells. Quantiferon-TB gold in the tube (Cellestis, Ltd., Carnegie, Australia) test was undetermined, while the tuberculosis skin test (TST) nonreactive. A broncoalveolar lavage (BAL) was performed, and microbiologic investigations resulted negative for bacteria and fungi including *P. jirovecii*.

**Figure 2:**
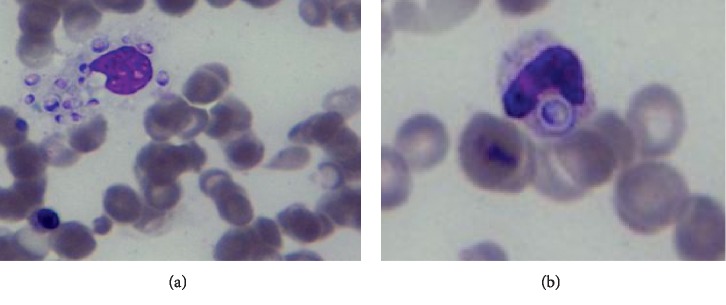
Bone marrow aspirate (a) and peripheral blood smear microscopy (b) showing yeast-like cells of *H. capsulatum* with a clear halo around them (Jenner–Giemsa stain; 1000x).

**Figure 3:**
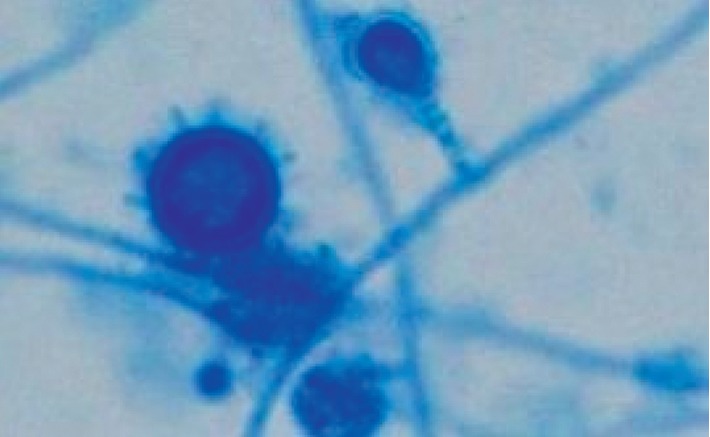
*H. capsulatum* mycelial phase macroconidia and microconidia (lactophenol cotton blue stain, 400x) from Sabouraud dextrose agar plates incubated at 30°C.

The patient was started empirically on sulfamethoxazole-trimethoprim (800/160 mg tid, oral), ceftriaxone 2 g I.V. daily, and foscarnet 90 mg/kg I.V. twice daily without improvement. Three days later, ceftriaxone was substituted with tigecycline. After 3 more days, both a bone marrow biopsy and a bone marrow aspirate were performed. The latter (see Figure 2(a)) revealed inclusion bodies inside the cytoplasm monocytes, suggestive of *H. capsulatum* yeasts. An identical finding was observed from the peripheral blood smear (see Figure 2(b)). In the following days, from both the peripheral vein blood and bone marrow aspirate, cultures on Sabouraud dextrose agar incubated at 30°C and grew *H. capsulatum* (Figure 3).

Liposomal amphotericin B 3 mg/kg/day was administered [6], while tigecycline was discontinued and sulfamethoxazole-trimethoprim dosage was reduced to 800/160 mg daily. Over the next 3 days, the skin lesions disappeared. A week after this, the fever persisted at 38°C, platelet count was 19 × 10^3^/*μ*L, haemoglobin was 6.6 g/dL, and the CMV-p65 antigen resulted negative without evidence of CMV organ localization. In light of this, foscarnet was discontinued and voriconazole 200 mg bid orally was added to amphotericin [3]. Two days later, the platelet count dropped to 8 × 10^3^/*μ*L, and at this point, amphotericin B was discontinued. The day after, the fever was resolved, while increases were observed for the platelet count (82 × 10^3^/*μ*L) and the haemoglobin value (10 g/dL) in the following 5 days. Voriconazole was administered for another 3 weeks and then substituted with itraconazole 200 mg tid for 3 days, followed by 200 mg bid as maintenance for 1 year. Antiretroviral therapy, including tenofovir disoproxil fumarate/emtricitabine and dolutegravir, was stared on the third week of antifungal treatment.

In 2017, one year after admission, CD4+ cell count was 207 cell/*μ*L (17%) and HIV-RNA was undetectable; itraconazole was discontinued.

In 2019, the patient is on the same ART therapy, the CD4+ lymphocytes are 808 cells/*μ*L (33.2%), and HIV-RNA remains undetectable.

## 3. Discussion

In Latin America, PDH is probably one of the most frequent AIDS-defining illness and the cause of AIDS-related deaths. Like in our patient, it often represents the first manifestation of an HIV infection [7]. A CD4 count less than 150 cells/*μ*L in persons living with HIV infection is the major risk factor for acquiring PDH [7, 8].

PDH is reported being the third opportunistic mycosis among Cuban persons living with HIV [9]. Nevertheless, it is an underdiagnosed disease, even in these endemic areas [2]. In Europe, including Italy, most of the cases are diagnosed among immunocompromised patients, either those with HIV infection or those who have visited endemic regions [3–5, 10, 11].

Symptoms of PDH start often within 2 months from inhalation of *H. capsulatum*; however, a latency up to 5 years has been also reported. A longer interval is more likely due to a reactivation of the disease concomitant to immune depression [9]. Our patient complained of fever 3 months after returning from Cuba, Camaguey Region, where the fungus is widespread [9]. It is likely that this patient returning to Italy presented a nonautochthonous PDH due to the reactivation of a latent infection triggered by the severe immune deficit status.

PDH clinical features are variables depending on cell-mediated immunity of the host, inoculum size, and duration of exposure [1, 12]. They are nonspecific, can mimic other etiologies including hematologic malignancy and hemophagocytic syndrome [13], and are responsible for delays in the diagnosis. PDH can also manifest skin involvement with several patterns like maculopapular lesions, pustules, nodules, ulcers, molluscum-like lesions, and keratotic plaques [14]. Skin lesions are reported in about 10% of patients with PDH from North America, while it can reach up to 93% in those from Latin America [14]. Some authors have explained the higher frequency of skin lesions in patients from Latin America as the result of a delay in the diagnosis in these countries, a different tropism of the fungus and/or a greater virulence [15]. Cutaneous manifestations appear later in the course of the disease and mostly in the disseminated form. The extension of the skin involvement can also correspond to the severity of the immunodeficiency [16]. Cuban epidemiology follows the rule, and papules are the most frequent features [17]. In our patient, clinical findings, including skin eruption and laboratory abnormalities, are similar to those reported in the literature [1, 4, 12].

With regard to diagnosis, the bone marrow aspirate and the peripheral blood smears microscopy have been proven to be effective for the diagnosis of *H. capsulatum* infection [1, 12, 18]. In our case, bone marrow biopsy allowed diagnosis and to rule out lymphoproliferative diseases and hemophagocytic lymphohistiocytosis [13]. Blood and urine *histoplasma* polysaccharide detection has also been indicated as a mainstay in the diagnosis of PDH and also is important to monitor treatment failure or relapses [6, 12]. The test is unavailable at our hospital.

Following current international guidelines [6], we started treatment with liposomal amphotericin B, achieving a reduction of fever. However, 10 days later, the fever persisted, and being so, voriconazole, with a higher bioavailability than itraconazole, was added to enhance antifungal activity of amphotericin [18–21]. After another 3 days, fever persisted and thrombocytopenia worsened, reaching 8 × 10^3^/*μ*L. Interpreting these manifestations as possible side effects of amphotericin B, it was discontinued [22, 23]. Treatment was completed with oral voriconazole. Overall, the infection was controlled: thereafter, the patient was maintained on suppressive therapy with itraconazole for one year [6, 17].

Some authors have reported a beneficial effect of sulfamethoxazole-trimethoprim in patients with PDH, suggesting that it could be a marker of access to care or an added therapeutic advantage of sulfa drugs for histoplasmosis. These drugs were used in the past for treatment of histoplasmosis [24]. In our patient, sulfamethoxazole-trimethoprim was administered for only 6 days before diagnosis was reached, and therefore, it is not possible to draw any conclusions on its role.

With regard to HIV infection, our patient matched the characteristics of age (33-year-old) and risk behavior (unprotected heterosexual intercourses), characterizing the epidemiology of HIV infection in her native country [25]. We believe the patient had been HIV infected for years prior to our diagnosis of PDH.

About the timing of ART initiation, as immune reconstitution inflammatory syndrome (IRIS) is reportedly uncommon in HIV-infected patients with histoplasmosis, ART should not be withheld because of concern for the possible development of IRIS [6, 26, 27]. Due to the poor condition of the patient, ART was initiated 3 weeks after starting antifungal therapy.

The prescribed antifungal therapy was effective, and ART has allowed an immune viral control, which is essential for reducing the risk of *H. capsulatum* reactivation [28].

## 4. Conclusions

*H. capsulatum* infection is a rarely diagnosed disease in Italy.

This case report highlights the need to suspect histoplasmosis in the differential diagnosis of opportunistic infections in immunocompromised persons native to or who have traveled to endemic countries. An update on PDH treatment protocols is needed.
